# Identification and Comparative Analysis of Differential Gene Expression in Soybean Leaf Tissue under Drought and Flooding Stress Revealed by RNA-Seq

**DOI:** 10.3389/fpls.2016.01044

**Published:** 2016-07-19

**Authors:** Wei Chen, Qiuming Yao, Gunvant B. Patil, Gaurav Agarwal, Rupesh K. Deshmukh, Li Lin, Biao Wang, Yongqin Wang, Silvas J. Prince, Li Song, Dong Xu, Yongqiang C. An, Babu Valliyodan, Rajeev K. Varshney, Henry T. Nguyen

**Affiliations:** ^1^Division of Plant Sciences, University of MissouriColumbia, MO, USA; ^2^Department of Computer Science and Christopher S. Bond Life Sciences Center, University of MissouriColumbia, MO, USA; ^3^Center of Excellence in Genomics, International Crops Research Institute for the Semi-Arid TropicsHyderabad, India; ^4^Legume Biotechnology Laboratory, School of Agriculture and Biology, Shanghai Jiao Tong UniversityShanghai, China; ^5^Plant Genetics Research Unit, Donald Danforth Plant Science Center, US Department of Agriculture, Agricultural Research Service, Midwest AreaSt. Louis, MO, USA

**Keywords:** RNA-Seq, soybean, drought, flooding, stress, gene expression

## Abstract

Drought and flooding are two major causes of severe yield loss in soybean worldwide. A lack of knowledge of the molecular mechanisms involved in drought and flood stress has been a limiting factor for the effective management of soybeans; therefore, it is imperative to assess the expression of genes involved in response to flood and drought stress. In this study, differentially expressed genes (DEGs) under drought and flooding conditions were investigated using Illumina RNA-Seq transcriptome profiling. A total of 2724 and 3498 DEGs were identified under drought and flooding treatments, respectively. These genes comprise 289 Transcription Factors (TFs) representing Basic Helix-loop Helix (bHLH), Ethylene Response Factors (ERFs), myeloblastosis (MYB), No apical meristem (NAC), and WRKY amino acid motif (WRKY) type major families known to be involved in the mechanism of stress tolerance. The expression of photosynthesis and chlorophyll synthesis related genes were significantly reduced under both types of stresses, which limit the metabolic processes and thus help prolong survival under extreme conditions. However, cell wall synthesis related genes were up-regulated under drought stress and down-regulated under flooding stress. Transcript profiles involved in the starch and sugar metabolism pathways were also affected under both stress conditions. The changes in expression of genes involved in regulating the flux of cell wall precursors and starch/sugar content can serve as an adaptive mechanism for soybean survival under stress conditions. This study has revealed the involvement of TFs, transporters, and photosynthetic genes, and has also given a glimpse of hormonal cross talk under the extreme water regimes, which will aid as an important resource for soybean crop improvement.

## Introduction

Soybeans, the most important legume crop worldwide, are an essential source of oil and protein for humans and livestock, and are also considered a potential source of bio-diesel (Koberg et al., 2011). Considering the importance of soybeans for food and nutritional security, there have been extensive efforts toward increasing soybean production. However, due to increases in the global population and its demand, there is a need for enhancing soybean productivity and production. Despite extensive efforts, soybean yield improvement is facing severe challenges and suffers with yield loss due to a range of biotic and abiotic stresses. Global climatic changes also play a vital role in influencing the abiotic and biotic conditions. Among abiotic stresses, extreme water regimes such as drought and flooding cause severe yield losses in all major crops including soybeans, rice, corn, and wheat (Perata et al., 2011). Drought is caused by insufficient water supply either from rainfall or groundwater, and results in the soil drying. In contrast, flooding is caused by heavy rainfall and results in water logging and submergence. In soybeans, both drought and flooding can cause up to a 40–60% yield loss worldwide (Valliyodan and Nguyen, 2006; Komatsu et al., 2009; Ahmed et al., 2013).

The mechanism that overcomes drought stress in plants is facilitated by drought avoidance, drought tolerance, drought escape, and drought recovery (Cruz De Carvalho, 2008). Drought causes a negative impact on all developmental stages, starting from germination to seed maturation (Valliyodan and Nguyen, 2006). Flooding also leads to very sensitive responses that affect both soybean growth and yield (Komatsu et al., 2009). Stress-specific adaptive mechanisms and various molecular, biochemical, and physiological responses in plants help maintain normal growth and survival under drought and flooding conditions (Dat et al., 2004). The perception of the environmental signal and subsequent molecular signaling is the first step in a stress response. In this direction, several signal compounds including abscisic acid (ABA), have been identified and studied for drought stress. The ABA level is up-regulated under drought conditions and the downstream responsive network is induced (Xiong and Zhu, 2002). The initiation of the ABA signaling process involves PYR/PYL/RCAR (Pyrabactin Resistance /PYR1-Like/Regulatory Components of ABA Receptors) ABA receptors, and two enzymes, protein phosphatase 2c family (PP2Cs) and SNF-related kinase (SnRK2), with opposite functions of phosphatase/kinase (Park et al., 2009; Kim, 2014). The binding of ABA to PP2Cs can inhibit PP2C activity, which causes it to lose its ability to inhibit SnRK2s. Then, activated SnRK2s can phosphorylate and activate the downstream ABFs (ABA responsive element binding factor), which can bind to their own targets (or other genes) and activate the downstream response pathways (Park et al., 2009). This causes plant to express a drought tolerance phenotype. The ABA level is also increased in flooding treatments in tomato (Else et al., 1995). However, the ABA level is reduced under flooding treatment in rice seedlings during submergence (Saika et al., 2007). Among different pathways of stress response signaling, most of the ABA dependent and independent responses involve TFs. These TFs belong to very diverse families representing ~10% of the genes in soybean, which are involved in most of the biotic and abiotic stress responses (http://planttfdb_v1.cbi.pku.edu.cn:9010/web/index.php?sp=gm). In rice, SUBMERGENCE1 (*SUB1*) has been identified as an important TF involved in submergence tolerance (Xu et al., 2006).

Antioxidants, such as ascorbate, glutathione, and tocopherol, are accumulated to protect the plants against reactive oxygen species (ROS), which are over-generated under drought and flooding conditions (Valliyodan and Nguyen, 2006; Chen et al., 2015). Photosynthesis is the major producer of ROS that enables the chloroplasts either to avoid its production or process it in the antioxidant network (Foyer and Shigeoka, 2011). Photosynthetic activity is reduced and carbohydrate metabolism changes under drought stress (Tabaeizadeh, 1998; Krasensky and Jonak, 2012; Chen et al., 2015). Similarly, ROS production increases due to heat, pathogen invasion, wounding, and low oxygen, while it decreases due to low light conditions that may arise during submergence (Steffens et al., 2013).

The complex molecular mechanisms, signaling perception, integrated responses, and molecular cross talk activated in response to different abiotic stress are not well-understood in soybean. In this regard, several efforts have been made to elucidate the molecular mechanisms. Genome-wide transcriptome profiling will be helpful in understanding a global view of gene expression under drought. Recently, drought-responsive candidate genes (*GmNAC*) have been identified using transcriptome profiling of soybean under drought conditions performed with a 66K Affymetrix microarray platform (Le et al., 2012). It has been reported that transcription profiles of the cold- and dehydration-responsive genes were similar among Arabidopsis, rice, and soybean, which shows representative up-regulated late embryogenesis abundant (dehydrin/LEA) and down-regulated (photosynthesis-related) genes. This suggests that different species have conserved different stress responses (Maruyama et al., 2012). Similarly, several studies using microarray platforms have been conducted and expression data for over 5000 soybean samples have been deposited in a public database (http://www.ncbi.nlm.nih.gov/geo/). However, the microarray have some drawbacks such as cross-hybridization, non-specific hybridization, and a limited sensitivity. Most importantly, microarray platforms provides the information on only those genes that are available on the microarray, which is a problem for soybean whose gene models are not well-characterized. These limitations can be overcome using recently advanced sequencing-based techniques, such as RNA sequencing (RNA-Seq; Varshney et al., 2009; Trapnell et al., 2013). In soybean, RNA-Seq has been deployed to generate an expression atlas for soybean genes using several tissues in seed development stages (Severin et al., 2010). In another study, variability in commercial and developing cultivars under drought conditions was identified using single nucleotide polymorphisms (SNPs) in RNA-Seq data (Vidal et al., 2012). In this study, genome-wide transcriptome profiling of soybean leaf tissue using the RNA-Seq approach was performed to understand the response to drought and flooding stress. The comparative analysis has enabled us to understand molecular responses against the extreme water availability conditions.

## Materials and methods

### Plant material and stress conditions

Soybean (William 82 genotype) plants were grown in 26.5 L pots with a dimension of 30 × 27 × 37 cm (top diameter × bottom diameter × height), filled with a mixture of turface and sand (2:1). Osmocote (Scotts Co., Marysville, OH) with a nutrient content of N:P_2_O_5_:K_2_O = 14:14:14 was added as a nutrient source at a rate of 20 g per pot. The plants were grown in a well-watered conditions [up to vegetative (V4) stage] under a 16 h photoperiod (~10,200 lux) at 28°C. Drought stress was imposed by withdrawing water for 7 days, while flooding stress was imposed by placing the pots into a bigger pot with a trashcan liner filled up to a water level of 4 cm above the soil surface for 7 days. After 7 days of treatment, all the leaves (irrespective of their response to stress) were sampled from the drought and flood treatments and control plants for RNA sequencing and real-time PCR analysis. The soil moisture was measured after the stress imposition in the drought experiment using a PR2 moisture probe (Delta T, UK).

### RNA isolation and library preparation

The total RNA was extracted using RNeasy Plant Mini Kit (Qiagen, Valencia, CA) and on-column DNase digestion was performed according to manufacturer's protocol. The concentration and quality of RNA were checked by a Nanodrop 1000 (Grand Island, NY) and by running the product on an agarose gel. The RNA-Seq libraries were prepared using a TruSeq Stranded mRNA LT Sample Prep Kit—Set A (Catalog #: RS-122-2101, Illumina, USA) according to the manufacturer's instructions. Three biological replicates were used for RNA-Seq and real-time PCR analysis.

### Quality filtering and mapping of RNA-Seq reads

The single end RNA-Seq reads were generated on the Illumina Genome Analyzer (San Diego, CA) platform. The processing of the initial reads was performed using the Illumina analysis pipeline (in the Fastq format) using custom and default parameters. Additional filtering was performed by removing adaptor sequences and low quality bases. To facilitate the read mapping, the *Glycine max* reference genome (Gmax1.1version) was indexed by Bowtie (http://www.phytozome.net; Langmead and Salzberg, 2012). The read mapping was performed using the Tophat software package (Trapnell et al., 2009; Kim et al., 2013). The reads were first mapped directly to the genome using indexing and then some of the unmapped reads were resolved by identifying novel splicing events. Two mismatched base pairs were allowed and the multiple position matching was reported up to 40 alignments using the Tophat mapping procedure. The transcriptome raw sequencing data from this study have been submitted on the NCBI (http://www.ncbi.nlm.nih.gov/) database as individual BioProjects: PRJNA324522.

### Sequence assembly and differential counting

The binary read alignment files were used as input to Cufflinks (Trapnell et al., 2009), which assembled the reads into transfrags (transcripts). The estimated gene abundance was then measured in terms of the fragments per kilobase of transcript per million mapped reads (FPKM). The differentially expressed genes (DEGs) between the two sets of samples were identified using cuffdiff. The significant up-regulated and down-regulated gene lists were obtained for the drought and flood samples, respectively. Only the genes with a log_2_ fold change ≥+2 and ≤ −2, but without infinite values and a FDR adjusted *p* ≤ 0.05 after Benjamini-Hochberg correction for multiple-testing with significance level “yes,” were considered as significantly DEGs.

### Functional annotation and gene ontology (GO) enrichment

The DEGs were annotated for gene ontology (GO) terms (Ashburner et al., 2000) and categorized into Molecular Function (MF), Cellular Component (CC), and Biological Process (BP) categories. A gene enrichment test was then performed on each of the gene lists to obtain the significant terms. Fisher's exact test, which is based on the hypergeometric distribution, was used to calculate the *p*-value. The TreeView (http://jtreeview.sourceforge.net/docs/overview.html) program was used to draw the heatmap of the significant DEGs in response to drought and flood stress.

### Pathways and TF identification and analysis

The drought and flood responsive pathways were identified and plotted using MapMan (Usadel et al., 2009). Multiple biological or metabolic pathways were plotted together with the mapped gene intensity of the fold change (≥ +2 and ≤ −2) by a blue and red schema. The TFs and transcription related genes were also mapped and plotted by MapMan. A more detailed TF family annotation was obtained from plantTFDB (Jin et al., 2014).

### Analysis of *Cis*-motif pattern

Using the collection of the motif sequences from the “Database of Plant *Cis*-acting Regulatory DNA Elements” (Higo et al., 1999), the 3 kb upstream of the 5′ translation start base was searched for all of the annotated soybean genes (phytozome v9.1). The number of genes was counted for each matched motif (gene count) and the total number of matches to all of the genes by each motif (hit count). Furthermore, counts from the subset of genes in each of the gene sets with all the genes were compared using Fisher's test. Any of the comparisons with a *p* ≤ 0.05 is presented in Supplementary Tables 3A,B.

### cDNA synthesis and qPCR

Total RNA was extracted from each sample using the Qiagen RNeasy mini kit (Qiagen, CA, USA).

The first strand cDNA from 1 μg of total RNA was synthesized using the EcoDry premix (Clontech, CA, USA), following the manufacturer's instructions. Quantitative PCR (qPCR) was performed using 10-fold diluted cDNA product in a 10 μL reaction volume using the Maxima SYBR Green/ROX qPCR Master Mix (Thermo, Waltham, MA, USA) on ABI7900HT detection system (Foster City, CA, USA). Three biological replicates and two technical replicates were used for analysis. The PCR was performed using two-step cycling protocol as follows: 50°C for 2 min; 95°C for 10 min, followed by 40 cycles of 95°C for 15 s, and 60°C for 1 min (https://www.thermofisher.com/order/catalog/product/K0221). To normalize the gene expression, Actin (*Glyma18g52780*) was selected as a housekeeping gene. All the primers were designed using QuantPrime software (http://www.quantprime.de) (Supplementary Table 5).

## Results

### Drought and flood treatment

Three biological replications were subjected to well-watered, drought, and flooding conditions at the V4 growth stage. Soil moisture, the major limiting factor used to impose drought stress on growing plants (Else et al., 1995), and was measured before and after the drought treatment. The soil moisture of the well-watered plant was 19% and was reduced to 10% after 7 days of drought treatment; the plants showed symptoms of leaf wilting. The degree of drought and flooding was determined by monitoring the canopy temperature. The canopy temperature (°F) increased from 76.8 ± 1.03 in normal conditions to 85.7 ± 4.42 and 79.4 ± 1.06 under the drought and flooding conditions, respectively.

### Mapping and differential gene expression analysis under drought and flooding stresses

The RNA samples from the soybean leaves under the control/drought/flooding conditions were used for sequencing by the Illumina Genome Analyzer. Approximately 28 million reads were generated from each sample (Table 1). The RNA-Seq analysis workflow is shown in Supplementary Figure 1 and was utilized for the data analysis. Approximately 72% of the reads were mapped to the soybean reference genome and ~2.5 to 4 million reads were mapped to multiple regions (Table 1).

**Table 1:** **Summary of RNA-Seq performed for soybean leaf tissue under drought and flooding stress**.

**Sample**	**Total Reads**	**Quality filtered reads**	**Uniquely mapped reads**	**Reads mapped to multiple locations**	**Mapping Percent**
Control_1	27,470,734	22,533,545	18,217,703	4,315,842	82
Control_2	29,705,720	21,254,999	17,447,699	3,807,300	72
Control_3	32,696,775	22,237,043	18,229,771	4,007,272	68
Drought_1	29,776,776	20,561,721	17,183,917	3,377,804	69
Drought_2	27,160,782	19,116,753	15,740,184	3,376,569	70
Drought_3	28,346,623	19,109,060	15,816,488	3,292,572	67
Flooding_1	25,775,222	18,061,225	15,149,722	2,911,503	70
Flooding_2	22,153,965	16,464,017	13,815,533	2,648,484	74
Flooding_3	28,307,353	20,646,668	17,299,235	3,347,433	73
Average	27,932,661	19,998,337	16,544,472	3,453,864	72

**Figure 1:**
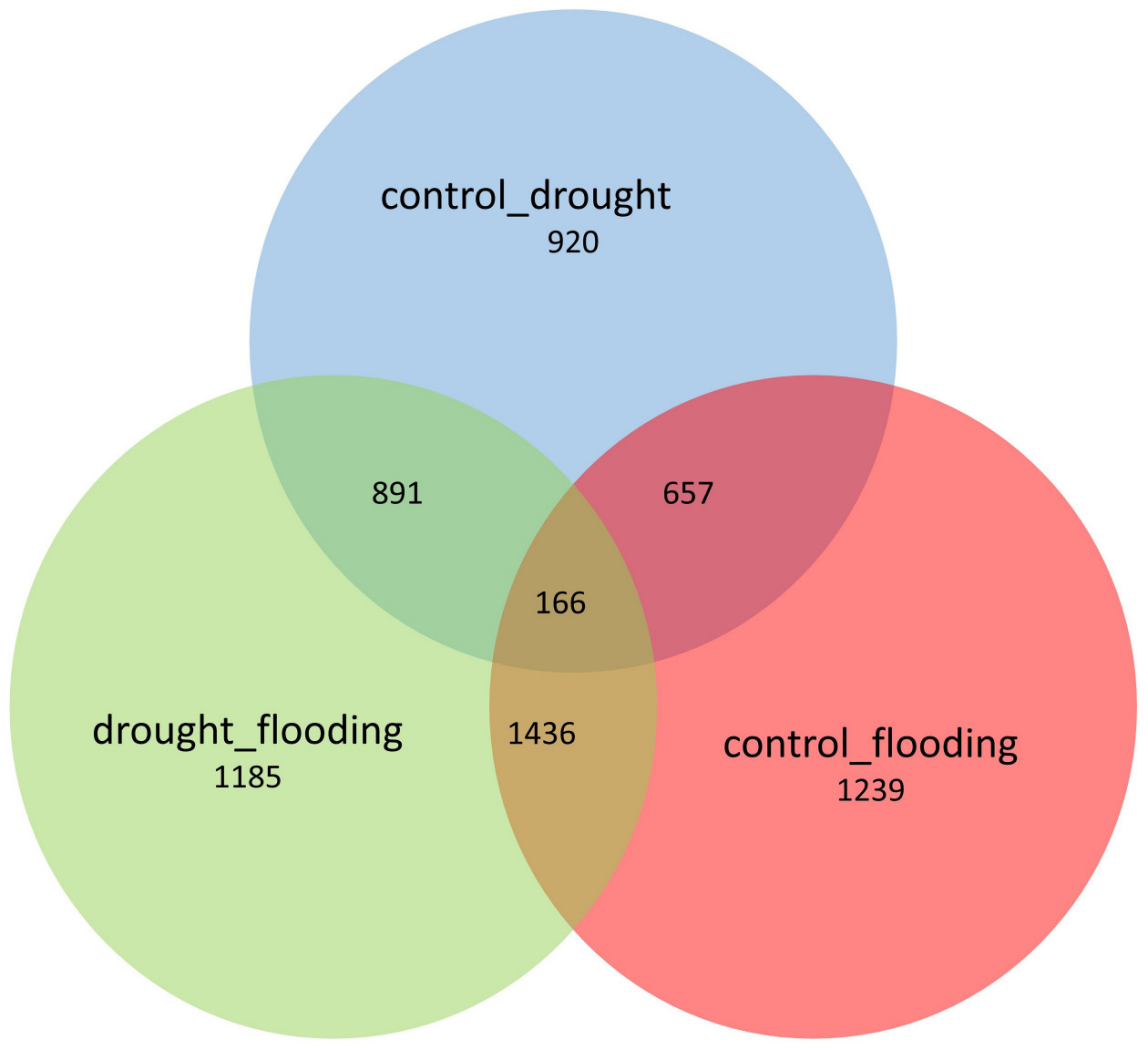
**Venn diagram representing the specific and common DEGs across the control vs. drought, control vs. flooding, and drought vs. flooding conditions**.

**Figure 2:**
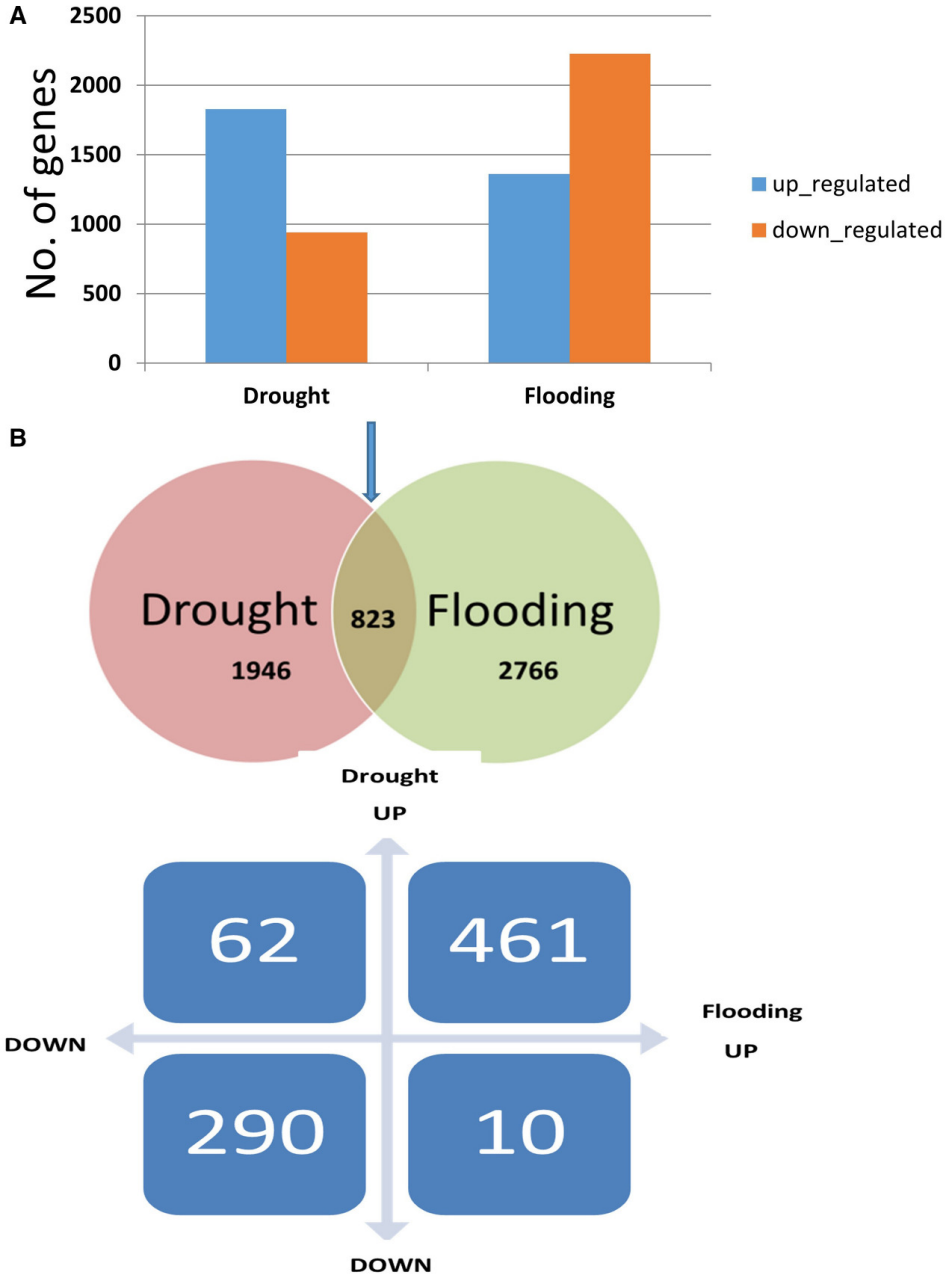
**The number of up- and down-regulated genes in drought and flooding conditions**. **(A)** Proportion of significant results (*p* ≤ 0.05, log_2_ fold change ≥2 for up-regulated and ≤ −2) for down-regulated genes in drought and flooding conditions. **(B)** The number of overlapped genes in two conditions.

To quantify and identify the genes, a core set of DEGs under drought and flood stress in soybean were examined. High-throughput RNA-sequencing analysis using Cufflinks pipeline (Trapnell et al., 2012) was performed in the following three combinations: (i) control vs. drought, (ii) control vs. flood, and (iii) drought vs. flood. The DEGs specific to and common between the stresses were also identified (Figures 1,2). A total of 36,968 and 37,434 genes with confident expression in drought and flood conditions, respectively, when compared to the control (Supplementary Tables 1A,B). Out of these genes, 36,239 were found to be common between the two conditions. The top 50 genes with >500 FPKM featured in the drought and flooding conditions were identified (Tables 2, 3). Interestingly, there are 15 genes among the top 50 highly expressed ones enriched for photosystem II (PS II) and dehydrin family proteins featured in both stress conditions. Flooding stress specific genes were found to be enriched for lipoxygenase 2, ethylene forming enzyme, matrixin family proteins, and 12-oxophytodienoate reductase 2, whereas drought specific genes were enriched for S-adenosylmethionine synthetase, calmodulin 5, 1-deoxy-D-xylulose-5-phosphate-reductoisomerase, and carbonic anhydrase (Tables 2, 3).

**Table 2:** **List of the 50 most highly expressed transcripts with their ontology and annotations in drought stressed leaf tissue compared to non-stressed control tissues**.

**Gene id**	**Transcript coordinate**	**FPKM**	**GO ids**	**GO type**	**Annotation**
Glyma01g04250	Gm01:3750043–3752379	1240.53	GO:0016758, GO:0008152	F,P	UDP-glucosyl transferase 74B1
Glyma02g47560	Gm02:51103817–51104907	1116.26	GO:0016020, GO:0009765	C,P	Photosystem II light harvesting complex gene 2.1
Glyma03g28760	Gm03:36707978–36709723	727.11			Dehydration-induced protein (ERD15)
Glyma03g32850	Gm03:40584884–40588047	1085.75	GO:0000902, GO:0005524	P,F	Heat shock cognate protein 70-1
Glyma03g38190	Gm03:44586431–44589302	682.88	GO:0004478, GO:0006556, GO:0005524	F,P,F	S-adenosylmethionine synthetase 1
Glyma04g01130	Gm04:710034–711409	1682.80	GO:0006950, GO:0009415	P,P	Cold-regulated 47
Glyma04g13490	Gm04:13109642–13110739	741.79	GO:0004857, GO:0030599	F,F	Plant invertase/pectin methylesterase inhibitor superfamily protein
Glyma05g13900	Gm05:14595247–14597672	916.44	GO:0005509, GO:0007165, GO:0005578	F,P,C	Calmodulin 5
Glyma05g25810	Gm05:31881851–31883064	1774.57	GO:0016020, GO:0009765	C,P	Chlorophyll A/B binding protein 1
Glyma05g28971	Gm05:34684777–34684906	1198.80			Conserved peptide upstream open reading frame 5
Glyma06g37260	Gm06:39710119-39711559	746.98			Metallothionein 3
Glyma07g01730	Gm07:1142812–1144391	2262.09	GO:0003993	F	HAD superfamily, subfamily IIIB acid Phosphatase
Glyma07g14340	Gm07:13708523–13710623	668.66	GO:0005509, GO:0015979, GO:0009523, GO:0009654, GO:0019898	F,P,C,C,C	Photosystem II subunit Q-2
Glyma07g35310	Gm07:40444012–40444809	2503.19			unknown function
Glyma08g08770	Gm08:6268663–6270567	1081.30	GO:0016020, GO:0009765	C,P	Chlorophyll A/B binding protein 1
Glyma08g18510	Gm08:13903708–13906551	1856.61	GO:0015979, GO:0009523, GO:0009654, GO:0042651	P,C,C,C	Photosystem II subunit R
Glyma09g15450	Gm09:17990279–17992556	657.97	GO:0006952, GO:0009607	P,P	MLP-like protein 34
Glyma09g36210	Gm09:42033362–42033506	928.14			ROTUNDIFOLIA Like 12
Glyma10g35871	Gm10:44076742–44077667	1098.55			Unknown function
Glyma11g18640	Gm11:15274089–15277211	690.62			Photosystem I subunit O
Glyma11g21616	Gm11:18750278–18750868	1923.28	GO:0005199	F	Unknown function
Glyma11g34230	Gm11:36096506–36100186	1518.22	GO:0005524	F	Rubisco activase
Glyma12g01120	Gm12:632601–632745	2063.58			ROTUNDIFOLIA like 12
Glyma12g05150	Gm12:3432975–3436225	850.75			Cold-regulated 413-plasma membrane 2
Glyma13g07610	Gm13:7807012–7808518	4066.37	GO:0016984, GO:0015977	F,P	Ribulose bisphosphate carboxylase (small chain) family protein
Glyma14g01130	Gm14:609754–611622	1241.04	GO:0016020, GO:0009765	C,P	Photosystem II light harvesting complex gene 2.1
Glyma14g06310	Gm14:4555027–4555301	1666.99	GO:0003735, GO:0006412, GO:0005840	F,P,C	Unknown function
Glyma14g09620	Gm14:7694931–7696850	813.56			Gibberellin-regulated family protein
Glyma14g26450	Gm14:32499281–32499395	1811.64			Rhodanese/cell cycle control phosphatase superfamily protein
Glyma15g04641	Gm15:3229565–3229843	1209.88			Unknown function
Glyma15g21890	Gm15:20279966–20282600	782.01	GO:0004478, GO:0006556, GO:0005524	F,P,F	S-adenosylmethionine synthetase family protein
Glyma15g38091	Gm15:44167213–44170746	664.74			Unknown function
Glyma15g40450	Gm15:47454286–47457571	896.08	GO:0015979, GO:0009523, GO:0009654, GO:0042651	P,C,C,C	Photosystem II subunit R
Glyma16g04190	Gm16:3559705–3561111	1309.08			Unknown function
Glyma16g04240	Gm16:3592381–3597724	922.43	GO:0003871, GO:0009086, GO:0008270, GO:0008652	F,P,F,P	Cobalamin-independent synthase family protein
Glyma16g04471	Gm16:3775380–3776171	963.52			Arabinogalactan protein 14
Glyma16g10880	Gm16:11230916–11238976	678.00	GO:0070402, GO:0055114, GO:0005515, GO:0046872, GO:0030604, GO:0008299	F,P,F,F,F,P	1-deoxy-D-xylulose 5-phosphate reductoisomerase
Glyma17g14481	Gm17:11219871–11220289	833.51			Sterol-4alpha-methyl oxidase 1-1
Glyma17g23900	Gm17:24100696–24103526	1014.65	GO:0003924, GO:0005525	F,F	GTP binding elongation factor Tu family protein
Glyma17g24193	Gm17:24549687–24552917	939.43			Dehydrin family protein
Glyma17g37760	Gm17:41489218–41490300	672.50			GAST1 protein homolog 1
Glyma18g04081	Gm18:2844663–2849361	1656.20	GO:0005524	F	Rubisco activase
Glyma19g01050	Gm19:738803–744963	1031.96	GO:0004089, GO:0008270, GO:0015976	F,F,P	Carbonic anhydrase 1
Glyma19g06370	Gm19:7281535–7282880	943.91	GO:0016984, GO:0015977	F,P	Ribulose bisphosphate Carboxylase (small chain) family protein
Glyma19g28240	Gm19:35628975–35631849	753.35	GO:0016620, GO:0055114	F,P	Glyceraldehyde 3-phosphate dehydrogenase A subunit 2
Glyma19g29210	Gm19:36800698–36804173	916.02			Unknown function
Glyma19g40810	Gm19:47126360–47129191	778.31	GO:0004478, GO:0006556, GO:0005524	F,P,F	S-adenosylmethionine synthetase 2
Glyma20g26871	Gm20:36185308–36186074	2273.27	GO:0003735, GO:0006412, GO:0005622, GO:0005840	F,P,C,C	Ribosomal protein S14p/S29e family protein
Glyma20g27950	Gm20:36940855–36943164	1158.99	GO:0005515	F	Polyubiquitin 10
Glyma20g28890	Gm20:37838080–37839638	706.21			Chlorophyll A-B binding family protein

**Table 3:** **List of the 50 most highly expressed transcripts with their ontology and annotations in flooding stressed leaf tissue compared to non-stressed control tissue**.

**Gene id**	**Transcript coordinate**	**FPKM**	**GO ids**	**GO term type**	**Annotation**
Glyma01g10070	Gm01:12864161–12867113	650.35			Conserved peptide upstream open reading frame 9
Glyma01g44600	Gm01:55217750–55219793	569.36	GO:0010181, GO:0016491, GO:0055114	F,F,P	12-oxophytodienoate reductase 2
Glyma02g03280	Gm02:2540711–2542352	2347.30			Unknown function
Glyma02g03301	Gm02:2556856–2568443	747.29	GO:0004222, GO:0008270, GO:0006508, GO:0031012, GO:0008152	F,F,P,C,P	Matrixin family protein
Glyma02g09220	Gm02:7198192–7198411	870.55			Unknown function
Glyma02g26160	Gm02:27007711–27014388	603.89	GO:0016702, GO:0046872, GO:0055114, GO:0005515	F,F,P,F	Lipoxygenase 2
Glyma02g43560	Gm02:48341044–48342999	1124.00	GO:0016491, GO:0016706, GO:0055114	F,F,P	Ethylene-forming enzyme
Glyma03g26060	Gm03:33395824–33397519	861.35	GO:0005507, GO:0009055	F,F	Uclacyanin 1
Glyma03g28850	Gm03:36781769–36785792	4180.10	GO:0004553, GO:0005975	F,P	Beta-1,3-glucanase 1
Glyma03g33340	Gm03:40943057–40946255	749.54			Glutathione S-transferase family protein
Glyma05g01845	Gm05:1304913–1306580	897.74			Gibberellin-regulated family protein
Glyma05g04490	Gm05:3644753–3645695	2133.60			Bifunctional inhibitor/lipid-transfer protein/seed storage 2S albumin superfamily protein
Glyma05g24110	Gm05:30116698–30119450	604.32	GO:0003924, GO:0005525	F,F	GTP binding elongation factor Tu family protein
Glyma05g27570	Gm05:33482002–33482310	881.32	GO:0003735, GO:0006412, GO:0005622, GO:0005840	F,P,C,C	Ribosomal L38e protein family
Glyma06g14820	Gm06:11615464–11617470	713.72	GO:0016872, GO:0042398	F,P	Chalcone-flavanone isomerase family protein
Glyma07g01730	Gm07:1142812–1144391	787.28	GO:0003993	F	HAD superfamily, subfamily IIIB acid phosphatase
Glyma07g15800	Gm07:15490518–15491924	1045.80	GO:0046872	F	Metallothionein 2A
Glyma07g35310	Gm07:40444012–40444809	740.77			Unknown function
Glyma08g18510	Gm08:13903708–13906551	789.50	GO:0015979, GO:0009523, GO:0009654, GO:0042651	P,C,C,C	Photosystem II subunit R
Glyma09g15450	Gm09:17990279–17992556	554.18	GO:0006952, GO:0009607	P,P	MLP-like protein 34
Glyma10g39780	Gm10:47393199–47395690	658.72	GO:0005515	F	Polyubiquitin 10
Glyma10g44370	Gm10:50779161–50779403	699.71			Unknown function
Glyma11g12505	Gm11:8920470–8921888	562.23	GO:0003676	F	Cold, circadian rhythm, and RNA binding 2
Glyma11g21616	Gm11:18750278–18750868	13673.00	GO:0005199	F	Unknown function
Glyma11g21633	Gm11:18768449–18769372	2884.8			Unknown function
Glyma13g07610	Gm13:7807012–7808518	1171.40	GO:0016984, GO:0015977	F,P	Ribulose bisphosphate carboxylase (small chain) family protein
Glyma13g20830	Gm13:24336368–24339828	634.18	GO:0003676	F	RNA-binding (RRM/RBD/RNP motifs) family protein
Glyma13g29330	Gm13:32257877–32258372	692.63	GO:0003735, GO:0006412, GO:0005622, GO:0005840	F,P,C,C	Ribosomal L38e protein family
Glyma13g43870	Gm13:43440262–43448189	564.06	GO:0005524, GO:0016887, GO:0016020	F,F,C	Pleiotropic drug resistance 12
Glyma14g03580	Gm14:2328701–2329110	1044.20			Unknown function
Glyma14g05650	Gm14:4071644–4072973	1258.40			Glycine-rich protein 3 short isoform
Glyma14g06310	Gm14:4555027–4555301	2247.20	GO:0003735, GO:0006412, GO:0005840	F,P,C	Unknown function
Glyma14g26450	Gm14:32499281–32499395	2203.50			Rhodanese/cell cycle control phosphatase superfamily protein
Glyma14g35823	Gm14:44952677–44952956	592.75			Conserved peptide upstream open reading frame 37
Glyma15g04641	Gm15:3229565–3229843	1084.60			Unknown function
Glyma15g08300	Gm15:5871359–5873073	912.29			Dormancy-associated protein-like 1
Glyma15g15200	Gm15:11629087–11631259	662.62	GO:0004553, GO:0005975	F,P	Glycosyl hydrolase superfamily protein
Glyma16g01500	Gm16:1083154–1086925	595.43	GO:0003700, GO:0006355	F,P	Related to AP2 2
Glyma16g05350	Gm16:4672459–4674745	2475.3			CCR-like
Glyma17g10050	Gm17:7524022–7526123	739.83			Gibberellin-regulated family protein
Glyma17g14481	Gm17:11219871–11220289	954.76			Sterol-4alpha-methyl oxidase 1-1
Glyma17g23900	Gm17:24100696–24103526	1126.8	GO:0003924, GO:0005525	F,F	GTP binding elongation factor Tu family protein
Glyma17g24193	Gm17:24549687–24552917	639.61			Dehydrin Family Protein
Glyma17g37760	Gm17:41489218–41490300	1486.7			GAST1 protein homolog 1
Glyma18g07520	Gm18:6230857–6233229	1574.9			Maternal effect embryo arrest 59
Glyma19g07240	Gm19:8549865–8552726	778.01	GO:0003924, GO:0005525	F,F	GTP binding elongation factor Tu family protein
Glyma19g27530	Gm19:34851350–34852853	1041.20			CCR-like
Glyma19g44916	Gm19:50221830–50222680	578.02	GO:0003735, GO:0006414, GO:0005622, GO:0005840	F,P,P,C	60S Acidic ribosomal protein family
Glyma20g26871	Gm20:36185308–36186074	3069.30	GO:0003735, GO:0006412, GO:0005622, GO:0005840	F,P,C,C	Ribosomal protein S14p/S29e family protein
Glyma20g27950	Gm20:36940855–36943164	1041.50	GO:0005515	F	Polyubiquitin 10

The distribution trends in terms of fold change ranged from ~8- to 9-fold change for DEGs under drought and flood stress (Supplementary Figure 2). A total of 2724 DEGs were identified under the drought conditions when compared to control, and 1802 genes were up-regulated and 922 genes were down-regulated (Supplementary Table 3A). During flood stress, a total of 3498 DEGs were identified when compared to the control, and 1303 genes were up-regulated and 2195 genes were down-regulated (Supplementary Table 2B). The total number of DEGs was greater in the flood condition compared to the drought. However, a greater number of up-regulated genes were observed under the drought condition compared to the flood condition with an overlap of 166 genes across the three comparisons (Figure 1). A total of 3768 genes were found to be differentially expressed when flood and drought conditions were compared to each other (Supplementary Table 2C). However, among the 2724 and 3498 DEGs identified in the drought and flood conditions, respectively, 823 DEGs were found to be in common (Figure 2A). Among these genes, 461 genes were both up-regulated under the drought and flood conditions, 290 genes were both down-regulated under the two conditions, 62 genes were up-regulated in the drought but down-regulated in the flood conditions, and 10 genes were down-regulated in the drought but up-regulated during flood conditions (Figure 2B).

### Gene ontology (GO) annotation of differentially expressed genes

**Figure 3:**
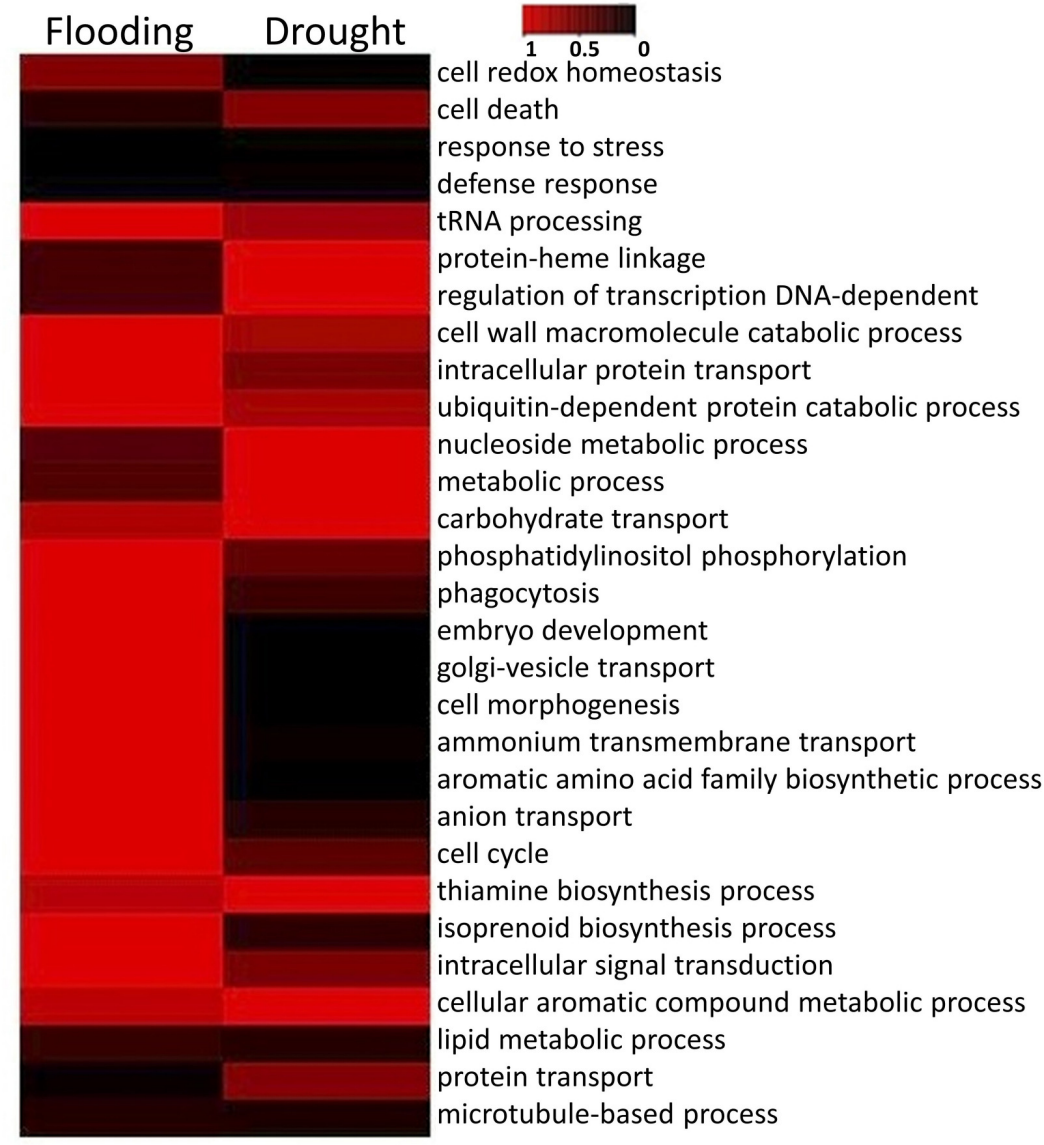
**The *p*-value heatmap of significant biological processes under drought and flooding conditions**. Darker color indicates greater significance.

The profiles of biological processes represented by GO enriched DEGs under drought and flood stresses were studied and represented (Figure 3, Supplementary Table 3). The heat-map revealed a stark difference in biological processes under different GO categories, such as phagocytosis, cell morphogenesis, cell cycle, isoprenoid biosynthesis, and transcriptional regulation represented by the enriched genes in these categories. However, little differences were observed in the profiles of the genes enriched for the GO categories “responsive to stress” and “defense response” under drought and flooding conditions. Biological processes such as “tRNA processing,” “cell wall macromolecule catabolic process,” “ubiquitin-dependent protein catabolic process,” and “carbohydrate transport” were reduced under both conditions (Figure 3). Most of the GO categories showed an opposite and comparable profile under drought and flood conditions, which suggested differential and specific gene regulation in both conditions. The GO categories of “cell redox homeostasis,” “intracellular protein transport,” “phosphatidylinositol phosphorylation,” “phagocytosis,” “embryo development,” and “Golgi vesicle transport” were enriched under flood condition but reduced under the drought conditions. On the contrary, “protein-heme linkage,” “regulation of transcription DNA-dependent,” “nucleoside metabolic process,” and “metabolic process” were enriched under drought but reduced under flooding conditions.

### Differentially expressed transcription factors (TFs) under drought and flooding stress

**Figure 4:**
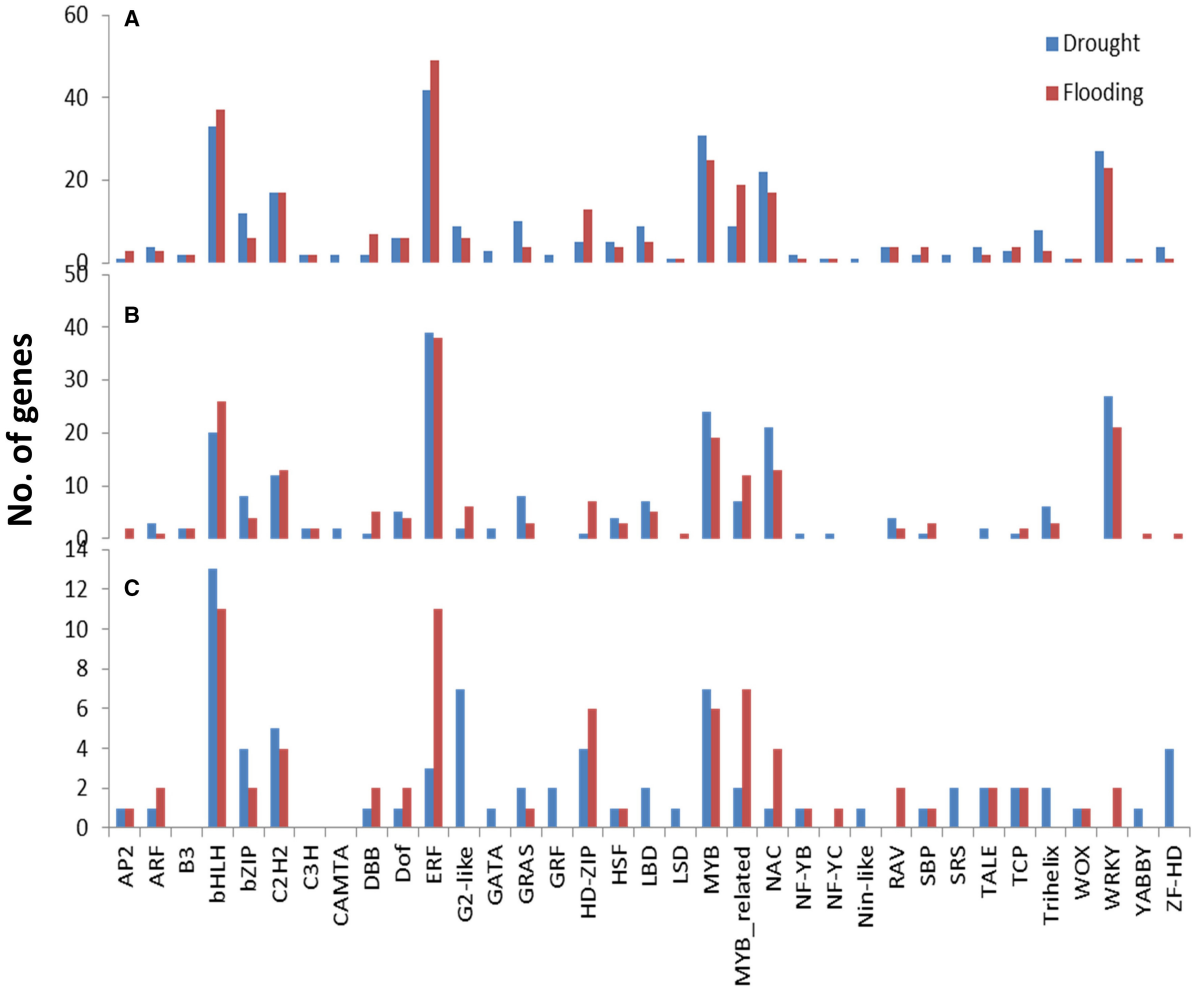
**Class-wise counts of transcription factors identified in DEGs under drought and flooding stress**. A total of 289 TFs (UP: 213, DOWN: 76) were found in drought and 271 TFs (UP: 199, DOWN: 72) in flooding stress. Identified from **(A)** all regulated genes, **(B)** only up-regulated genes, and **(C)** only down-regulated genes.

The RNA-Seq expression profiling revealed 289 and 271 differentially expressed TFs under drought and flood stress, respectively. A total of 213 and 199 TFs were up-regulated, whereas 76 and 72 TFs were down-regulated under drought and flood conditions, respectively (Table 4). Interestingly, the majority of differentially expressed TFs, irrespective of a family, were up-regulated under both conditions (Table 4). Genes belonging to the bHLH, ERF, MYB, NAC, and WRKY family represent most of the differentially expressed TFs. The Ethylene Response Factor (ERF) represented the highest number of significantly expressed genes under both drought and flooding conditions followed by bHLB, MYB, NAC, and WRKY (Table 4, Figure 4, Supplementary Figure 5).

**Table 4:** **Details of differentially expressed TFs identified by RNA-Seq and performed for drought and flooding conditions in soybean**.

**TF Class**	**All**		**Up-regulated**		**Down-regulated**	
	**Drought**	**Flooding**	**Drought**	**Flooding**	**Drought**	**Flooding**
AP2	1	3	0	2	1	1
ARF	4	3	3	1	1	2
B3	2	2	2	2	0	0
bHLH	33	37	20	26	13	11
bZIP	12	6	8	4	4	2
C2H2	17	17	12	13	5	4
C3H	2	2	2	2	0	0
CAMTA	2	0	2	0	0	0
DBB	2	7	1	5	1	2
Dof	6	6	5	4	1	2
ERF	42	49	39	38	3	11
G2-like	9	6	2	6	7	0
GATA	3	0	2	0	1	0
GRAS	10	4	8	3	2	1
GRF	2	0	0	0	2	0
HD-ZIP	5	13	1	7	4	6
HSF	5	4	4	3	1	1
LBD	9	5	7	5	2	0
LSD	1	1	0	1	1	0
MYB	31	25	24	19	7	6
MYB_related	9	19	7	12	2	7
NAC	22	17	21	13	1	4
NF-YB	2	1	1	0	1	1
NF-YC	1	1	1	0	0	1
Nin-like	1	0	0	0	1	0
RAV	4	4	4	2	0	2
SBP	2	4	1	3	1	1
SRS	2	0	0	0	2	0
TALE	4	2	2	0	2	2
TCP	3	4	1	2	2	2
Trihelix	8	3	6	3	2	0
WOX	1	1	0	0	1	1
WRKY	27	23	27	21	0	2
YABBY	1	1	0	1	1	0
ZF-HD	4	1	0	1	4	0
Total	289	271	213	199	76	72

### Photosynthesis associated responses

The photosynthesis related genes were down-regulated under both drought and flooding conditions. The expression level of three PS-II light harvesting complex (LHC) related genes, *Glyma16g28030.1, Glyma16g28070.1*, and *Glyma10g04630.1*, were repressed significantly under drought conditions. Similarly, three photosynthesis light reaction related genes, *Glyma07g21150.1, Glyma20g24500.1*, and *Glyma20g24500.1*, and one Calvin cycle related gene, *Glyma01g34300.1*, were also down-regulated under drought conditions. Only one gene from the photosynthetic system encoding an electron carrier was induced under the drought conditions (Supplementary Figure 6A). However, compared to the drought, more photosynthesis related genes were found to be down-regulated under flooding conditions (Supplementary Figure 6B). The relative expression level of 34 genes encoding PS-II LHC and 45 genes encoding PS-II subunits were repressed (Supplementary Figure 6B). In addition, 41 genes related to the Calvin cycle and eight electron carrier related genes were also repressed. In total, seven genes were down- and one was up-regulated under drought stress. However, 128 genes were down-regulated under flood treatment (Supplementary Figure 6B). The dynamic expression of photosynthesis related genes can be correlated with the chlorophyll content under drought and flood conditions, which concurs with the inhibition of photosynthesis (Supplementary Figures 3A,B).

### Sugar and starch metabolism pathways

Changes in the expression levels of a large number of sucrose and starch metabolism pathway genes were observed under the drought and flood conditions. Genes encoding sucrose and starch biosynthesis genes, namely, amylose synthase, ADP-Glucose synthase, and starch and maltose synthase, showed a significant decrease in the transcript level in drought and flooding treatments (Supplementary Figure 4). No change in expression was observed for sucrose phosphate synthase genes (*Glyma08g42140.1* and *Glyma06g48200.1*) in the drought condition, in contrast to the significant repression in expression of these genes under the flood conditions. However, the genes encoding beta-fructofuranosidase (*Glyma13g42530.1*), fructokinase (*Glyma16g32530.1*), hexokinase (*Glyma11g10130.1*), and three maltose synthase genes (*Glyma13g28630.1, Glyma11g04210.1*, and *Glyma15g10480.1*) were up-regulated under the drought conditions. Overall, transcript abundance for starch biosynthesis encoding genes was reduced more in the flooding stress compared to the drought stress. This could result in the decrease of starch content under flooding conditions (Supplementary Figure 4B). Only one maltose synthesis gene, *Glyma11g04210.1*, was induced during flooding (Supplementary Figures 4, 6). We also identified six genes of the soybean sugar-effluxer (SWEET) family that was differentially expressed under the two extreme water regimes. Three genes, *Glyma12g36300, Glyma14g27610*, and *Glyma06g13110*, were up-regulated under the drought conditions, and the other three genes, *Glyma06g17520, Glyma09g04840*, and *Glyma13g33950*, were down-regulated under flooding conditions. Interestingly, one SWEET gene, *Glyma14g27610*, was up-regulated in both drought and flooding conditions (Supplementary Tables 1A,B).

### Cell wall associated responses

The annotated RNA-Seq data was analyzed for the cell wall precursor's related genes. Expression of 11 cell wall precursor's related genes were up-regulated in the drought conditions, whereas not a single gene was observed to be down-regulated. On the contrary, the flooding condition showed a reduced transcript level for 10 cell wall precursor synthase genes and three genes were up-regulated (Supplementary Figure 7). The UDP-Glucosyl Transferase enzyme coding gene under drought stress was found to be highly expressed (FPKM = 1240.53; Table 2). This enzyme is known to be involved in the formation of β-glucans, which are thought to be involved in cell wall formation (Kosegarten et al., 1988).

### Analysis of *Cis*-motif pattern

Genes with similar expression patterns are likely co-regulated and have the same *cis*-regulatory elements (CREs or known as motifs; Priest et al., 2009). The set of DEGs (up- and down-regulated genes) identified under flood and drought treatments in this study was utilized (Supplementary Figure 2). Using the collection of motif sequences from the “Database of Plant *Cis*-acting Regulatory DNA Elements,” three kb upstream of the 5′ most translation start base for all of the annotated soy genes was searched (Phytozome v9.1). Sixteen and 76 over-represented motifs in the down- and up-regulated drought response genes, respectively, and 48 and 5 over-represented motifs in the down- and up-regulated flood response genes, respectively were identified (Supplementary Table 4A). No overlapping motifs were found among the drought and flood response up-regulated genes; however, eight overlapped motifs were found between the drought and flooding down-regulated genes. Additionally, no overlapped motifs were found between the drought down-regulated genes and flooding up-regulated genes, and 30 overlapped motifs were found between the up- and down-regulated drought and flood genes (Supplementary Table 4B). Among the 30 overlapped motifs, 24 showed the ACGT sequence, which serves as a binding site for the bZIP class of TFs. It has been reported that the flanking sequence of the ACGT core motif is responsible for differential distribution of the stress responsive regulatory elements in the stress responsive promoters (Suzuki et al., 2005). The nucleotides flanking the ACGT motif determine the binding affinity and specificity of bZIP TFs, which in turn leads to distinct physiological functions in stress responses, light regulation, etc. (Izawa et al., 1993; Foster et al., 1994).

Although drought stress up-regulated genes shared several *cis*-regulatory elements with down-regulated flood stress genes, few genes were found to be common between drought up-regulated and flooding down-regulated genes. For example, there are 324 up-regulated drought responsive genes and 457 down-regulated flood response genes with only one shared motif, ACGTABREMOTIFA2OSEM. Importantly, this motif acts at the core of ACGT of motif A in the rice ABRE gene (Hattori et al., 2002). However, there were only 16 common genes representing 5% of the drought up-regulated genes and 4% of the flood down-regulated genes.

Drought and flood down-regulated genes had ~15% of their genes in common (Supplementary Table 3D). Interestingly, flood down-regulated genes shared most their over-represented motifs (30/48) with drought up-regulated genes. However, they did not share many genes between the down-regulated flooding response and the up-regulated drought responsive genes (Supplementary Tables 3C,D). The molecular function categories were also examined using the GO enrichment analysis for both of the datasets represented by the ACGTABREMOTIFA2OSEM motif. Interestingly, the top three molecular functions for both of the datasets were “catalytic activity,” “sequence-specific DNA binding TF activity,” and “DNA binding.”

### Quantitative real-time PCR (qRT-PCR) validation of DEGs from RNA-Seq

**Figure 5:**
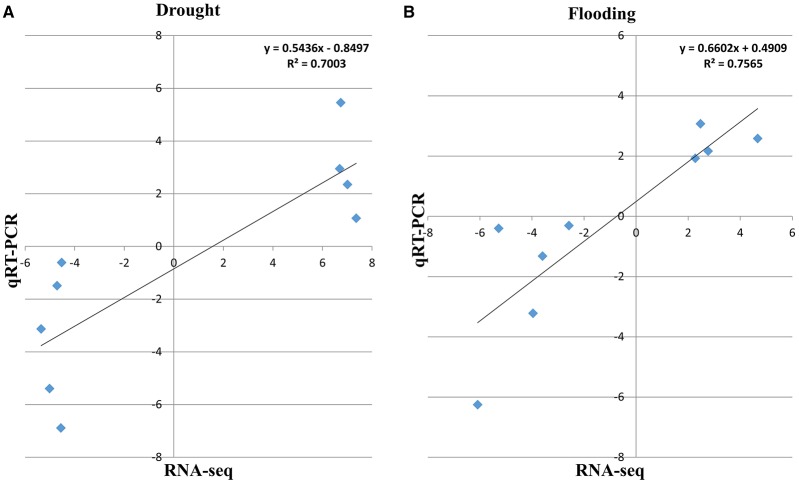
**The qRT-PCR validation of differentially expressed genes in soybean under drought and flooding conditions**. The correlation of the fold change analyzed by RNA-Seq (*x*-axis) with data obtained using real-time PCR (*y*-axis; **A)** under drought and **(B)** under flooding stress.

To validate RNA-Seq analysis, qRT-PCR was performed. A few key genes were selected based on increased or reduced transcript abundance under drought and flooding conditions. Although the RNA-Seq values showed slight variations compared with the corresponding values from the qRT-PCR analyses, the expression data and pattern from RNA-Seq were largely consistent with those obtained by qRT-PCR with a high correlation coefficient value (*R*^2^ = 0.70, 0.76 for drought and flood responsive genes, respectively; Figure 5).

## Discussion

The results presented here demonstrate the unique and differential response of soybean leaf tissue under two extreme water regimes, drought and flood. Several 100 genes are known to be differentially expressed under abiotic stresses such as drought, high salinity, low temperature, and flooding (Deshmukh et al., 2014; Patil et al., 2016). In addition to the involvement of various physiological and molecular mechanisms, most of the pathways and genes are found to be common across the different stress responses (Deshmukh et al., 2014). Transcriptome profiling by RNA-Seq has enabled comparison of a transcriptional response under drought and flooding stress in soybeans, and also development of a catalog of DEGs. The direct comparison of genes expressed under different conditions or experiments would require a meta-analysis (Bhargava et al., 2013) to have a better insight into the modus-operandi of genes specifically and commonly involved in various stress responses. Nevertheless, the ratio of up- and down-regulated genes under two extreme water regimes in this study could be compared and ultimately shed some light on the comparative transcriptional response. However, a higher number of DEGs were identified under flood conditions, and the number of up-regulated genes was much higher under drought conditions (Figure 2). Similarly, fewer up-regulated genes under flood conditions in soybean have been reported by Nanjo et al. (2011). In agreement with Nanjo et al. (2011), we observed the lowered metabolic activity under flooding as compared to the drought, which was evident from the global gene expression profiling under the two stresses.

### Transcription factors regulation under drought and flooding stress in soybean

The involvement of TFs was examined in the drought and flood stress responses through both the ABA dependent and independent pathways. The ABA and other upstream signals can trigger the downstream regulation pathway through the TF regulatory network in both drought and flood conditions. In this research, ABA related TFs: AP2, bZIP, MYB, and NAC were up-regulated under both drought and flood conditions (Figure 4). These TF families have been extensively studied in many plants under several different stress conditions (Dubos et al., 2010; Hao et al., 2011; Mizoi et al., 2012; Nakashima et al., 2012,). In several abiotic stress regulation studies, the TFs identified are initially involved in tolerance against a specific stress and later observed to be important for other stresses. For instance, the *SUB1A* TF was initially identified as a key regulator for the flood responsive pathways in rice, and later transcriptional and functional studies confirmed its important role in drought tolerance (Xu et al., 2006; Fukao et al., 2011). However, the flood response in soybean seems to be independent of the *SUB1* pathway since no *SUB1A* homolog was found to be differentially expressed in our study. On the other hand, recent studies have shown the involvement of *SUB1* and core-clock related genes mediated through alternatively spliced forms (Syed et al., 2015).

### Hormonal signaling and transcriptional regulations under the extreme water regime

Genes including TFs involved in the ABA dependent stress response were found to be differentially expressed under both drought and flood stress conditions (Table 4, Supplementary Table 2). Although ABA is the most specifically studied hormone for its regulatory role in different stress conditions, other hormones such as cytokinins, brassinosteroids, auxins, and ethylene also played an important functional and regulatory role (Peleg and Blumwald, 2011; Patil and Nicander, 2013). The highest number of TFs belonging to the ERF family coincided with the higher number of ethylene related genes observed under drought and flooding conditions (Table 4). This suggests the role of ethylene in both drought and flood responsive pathways. Ethylene biosynthesis has been found to be affected by auxin, particularly with the auxin dependent roles of several members of the 1-aminocyclopropane-1-carboxylate synthase (ACS) family genes (Nakatsuka et al., 1998). S-Adenosylmethionine synthetase (SAM synthetase) coding genes were found to be highly enriched and featured among the top 50 highly expressed genes under drought stress. SAM acts as a precursor for polyamines and ethylene biosynthesis, which indicates a high efflux of ethylene hormone under stress to counteract the adverse conditions. Higher ethylene levels are known to induce the ethylene signaling cascade, which in turn involves the ERF TF activation. That is also found to be highly expressed under the stresses imposed in the current study. Higher ethylene levels are manifested as the ethylene triple response (Knight et al., 1910) to prevent root and shoot elongation, radial swelling of the root and shoot, and horizontal growth of the embryonic leaves and meristem instead of vertical growth (a consequence of auxin imbalance across the stem axis). This triple response prevents the plants from damage. It would also be interesting to see the receptor response against elevated ethylene levels as reviewed by Agarwal et al. (2012) because different plants perceive ethylene in their own unique way and the receptors have a very important role in signal perception and the downstream cascade.

The 1-Deoxy-D-xylulose-5-phosphate reductoisomerase (DXR) is an important enzyme involved in the 2-C-methyl-D-erythritol-4-phosphate (MEP) pathway for isoprenoid biosynthesis and was identified among the highly expressed drought responsive genes (Table 2). Xing et al. (2010) reported that *dxr* mutants with a dwarf phenotype significantly reduced the number of trichomes and abnormal stomatal closure, which was rescued with exogenous application of ABA and gibberellic acids (GAs). Thus, the high expression of DXR under drought stress implies a plant's response to withstand stress and compensate for compromised photosynthetic capacity and abnormal stomatal conductance. Auxin, ethylene, and ABA were previously identified to be involved in the drought responsive pathway in soybean (Le et al., 2012). Additionally, ethylene, ABA, and GA are known to be involved in a regulatory network under flooding stress (Bailey-Serres et al., 2012). Hormonal cross-talk at different levels of the stress response and the resultant synergetic or antagonist interactions have been found to be important for stress tolerance (Chinnusamy et al., 2004; Peleg and Blumwald, 2011). The biosynthesis of jasmonic acid (JA) through the octadecanoic pathway (Vick and Zimmerman, 1984) with α-linolenic acid (LA) as a precursor, is mediated by 12-oxophytodienoic acid reductase 2 (OPDAR2) (*Glyma01g44600*), which is found to be up-regulated under both drought and flooding stress (Supplementary Tables 1B, 2A) but highly expressed under flooding stress (Table 3). The high expression of OPDRA2 is correlated with higher JA synthesis and other systemic responses. The enzyme has also been shown to detoxify 2,4,6-trinitro toluene (TNT) in polluted water contaminated by explosives as a result of manufacturing and munitions (Beynon et al., 2009). The upsurge of OPDAR2 under flooding stress suggests plants' adaptive response against stress and this could also help in detoxification of contaminants such as TNT if any are present in the water. DEGs involved in different hormonal pathways identified here under drought and flooding stress could be good candidates to manipulate hormonal cross-talk for the effective regulation of stress tolerance in plants.

### Transcriptional response related to photosynthesis efficiency and chlorophyll

The genes involved in photosynthesis were found to be preferentially down-regulated under both drought and flood conditions in this study (Supplementary Figure 6). This agrees with the hypothesis that a plant's photosynthetic capacity is affected under stresses such as drought and flooding stresses. Thus, this implies that the reduction in photosynthesis activity is the major effect of drought and flooding in soybean. Similarly, the down-regulation of genes involved in photosynthesis has been previously reported under a water deficit situation in other crops (Wang et al., 2011). Interestingly, Nanjo et al. (2011) reported the up-regulation of photosynthesis related genes under flooding; however, they imposed stress at the 2 day-old seedling stage of soybean and performed transcriptome profiling with hypocotyl tissue. Similarly, during the early stress response in Arabidopsis (low oxygen, 3 h) and gray poplar (flooded 5 h), the up-regulation of photosynthesis related genes was observed (Kreuzwieser et al., 2009). In our study, the down-regulation of photosynthesis related genes was observed after 7 days of flood treatment (Supplementary Figure 6). The down-regulation of photosynthesis related genes has also been reported in the leaves of rape seedlings waterlogged for 3 days (Lee et al., 2014). This suggests that the flooding or low-oxygen stress stimulates the expression of photosynthesis related genes during the initial stress response but it is repressed at later stages. Flooding results in reduced photosynthesis manifested by depleted carbon dioxide (CO_2_) concentration in leaves mediated by the stomatal constraint to the exchange gases (Bailey-Serres et al., 2012). Reduced CO_2_ levels lead to a depleted electron sink in chloroplasts, which ultimately results in accumulation of photons that are a potential source of ROS. The shrunken electron sink under flooding conditions is also substantiated in our findings and is demonstrated as the repressed expression of eight electron carriers and PS II related genes (Supplementary Figure 6B). The chlorophyll synthesis related genes were found to be repressed under both drought and flooding conditions in this study (Supplementary Figure 3). The lower availability of oxygen (O_2_) and CO_2_ under flooding caused by the low gas exchange ratio (Bailey-Serres et al., 2012) may result in decreased photosynthesis efficiency and carbohydrate content. This was consistent with the reduced expression level of starch synthesis genes in the present study (Supplementary Figure 4).

Reduced photosynthesis during prolonged abiotic stresses such as drought and flooding can be correlated with the production of ROS (Klok et al., 2002). High levels of ROS can damage the cell through peroxidation of the lipids, oxidation of proteins and other pathways, and finally the death of the cell (Clement et al., 2008; Wrzaczek et al., 2011). Glutathione S-transferase, one of the important enzyme in the regulation of ROS flux, is featured as a highly enriched gene among the top 50 highly expressed genes under flooding stress in this study, which indicates another adaptive response under the stress conditions (Table 3). However, the opposite (high GST expression in response to drought compared to flooding stress) was reported by Oh and Komatsu (2015).

Drought also has severe effects on the stomatal conductance and low CO_2_ assimilation. It is often considered to be one of the most prominent reasons for poor photosynthesis. However, it is not the only reason. Another factor is reduced ATP synthesis leading to depleted ribulose 1,5-bisphosphate content (Tezara et al., 1999), which produces a 6C compound when combined with CO_2_ in the dark reaction (Calvin cycle) of photosynthesis. The repressed expression levels of genes associated with the Calvin cycle in this study (Supplementary Figure 6A) substantiates the decreased photosynthetic capacity mediated by down-regulated dark reaction genes. However, the observed up-regulated electron carrier related gene in drought stressed plants needs to be justified when the Calvin cycle and LHC related genes are down-regulated. This is because under lesser CO_2_ assimilation in drought stress, the photosynthetically accumulated energy becomes excess for the dark reaction, and to counteract this condition, over reduction of the electron transport chain occurs (Biehler and Fock, 1996).

### Transcriptional response toward cell wall plasticity under drought and flooding

Water stress can change the turgor pressure of the plant cell and it responds accordingly, either by tightening or loosening the wall under the extreme water conditions of drought or flooding conditions (Moore et al., 2008). UDP-glucuronic acid is the major precursor of cell wall polysaccharides and comes from two sources: UDP-glucose and myo-inositol (Siddique et al., 2013). The genes involved in both UDP-glucuronic acid synthesis pathways were up-regulated under drought stress (Supplementary Figure 7A). This can cause the accumulation of cell wall precursors and the increase of cell turgor under drought conditions. However, an adverse result was observed under flooding. The GDP-glucuronic acid synthesis genes were reduced under flooding (Supplementary Figure 4). This implies the loosening of the cell wall and stimulation of shoot elongation under flooding (Voesenek et al., 2006).

### Regulation of sucrose and starch synthesis under drought and flood conditions

Most research on the effect of drought and flooding on sugar metabolism and starch biosynthesis has demonstrated that the carbohydrate levels are altered in leaves (Lemoine et al., 2013). Under stress conditions, the increase in hexose amounts is associated with the induction of starch hydrolysis (Pelleschi et al., 1997). In agreement with these findings, we observed an increased expression of the hexokinase (*Glyma11g10130*.*1*) gene in the drought treatment. The gradual slowing down of starch metabolism is a general mechanism under flooding (Fukao et al., 2011) and drought (Burke, 2007) conditions for rice and cotton, respectively, which reserves the energy source for a prolonged energy supply to maintain cell survival. It is conceivable that starch metabolism during flooding and drought response may also be controlled/gated by the up- and down-regulation of carbohydrate metabolism genes in soybean. One of the prominent processes under sugar metabolism that gets affected in water stressed plants is the sugar efflux rate, which is carried out by proteins coded by sugar transporter genes. They ensure sugar transport from the source (leaves) to the sink (roots and seeds) to counteract the depleted sugar levels under severe conditions such as drought and flooding. Utilizing the available resource generated by Patil et al. (2015), six SWEET family genes were featured among the DEGs identified in this study under drought and flood conditions. The findings suggest that SWEET genes in general are induced in drought conditions and repressed in flooding conditions, which indicate that sucrose efflux genes are activated under drought conditions and repressed under flooding conditions. However, one gene (*Glyma14g276210*) was found to be up-regulated under both of the conditions. Syed et al. (2015) also identified the up-regulation of soybean SWEET genes under drought stress. The activated gene expression under drought stress could be accounted for by sugar transportation due to an uninterrupted energy supply under such adversity. The down-regulation of three SWEET family genes under flooding stress in the current study is contrary to the findings of Lemoine et al. (2013), where it was suggested that the up-regulated sugar effluxer genes may contribute to sugar homeostasis in a flood tolerant genotype. In this study, the repressed genes might be playing the same role by controlling the efflux of sugar under flooding stress and thus putting a check on normal efflux of sugar. However, this hypothesis needs additional substantiation.

Glycolytic enzymes along with inducers of heat shock proteins are key factors in the early response to flooding and drought in soybean (Nanjo et al., 2011; Oh and Komatsu, 2015), which suggests the significance of the glycolytic pathway in the adaptation to flooding and drought conditions. Oh and Komatsu (2015) reported increased expression of all glycolysis related proteins under both drought and flooding responses. The up-regulated expression of genes coding for the glycolytic pathway enzymes, fructokinase (*Glyma16g32530.1*) and hexokinase (*Glyma11g10130.1*), under drought stress can be considered an adaptive response of soybean under drought stress. On the contrary, the transcript levels of glycolytic pathway enzymes were not significantly affected under water deficit conditions in chickpea (Khanna et al., 2014). This suggests a legume specific adaptive variability under similar stress.

### *Cis*-regulation during drought and flooding treatments

The analysis of *cis* regulatory elements showed that although the genes between drought up-regulated and flooding down-regulated DEGs shared a common motif, they might have a similar function with opposite regulation under contrasting stress treatments. It has been reported that proteins with up to 67% amino acid sequence similarity may share similar high-affinity binding sequences, and prefer different low-affinity sites. However, even closely related TFs may have distinct DNA-binding profiles (Badis et al., 2009). The positions of the flanking sequence of motif sites also play an important role in achieving regulatory specificity within the TF families (Ciolkowski et al., 2008; Gordan et al., 2013). Therefore, it is possible that this opposite regulation between drought up-regulated genes and flooding down-regulated genes with the same motif could be due to the different TF binding sites or because of the different site specific cofactors with the same affinity for the same TF binding sites, which leads to activation or repression of the target genes.

## Conclusion

Plant responses toward abiotic stress are complex and involve several different mechanisms regulated by crosstalk between genes related to hormonal signaling, photosynthesis, respiration, and transcriptional regulations. Comparative transcriptome profiling performed in this study under drought and flooding has provided an opportunity to evaluate the categorized molecular responses. Besides having contrasting stress conditions, many common molecular mechanisms were observed to be involved under drought and flooding. A notable example is the ABA related TFs that are up-regulated under both drought and flood conditions. ABA is considered as the stress related hormone and seems to be a key regulator under both conditions. Unlike rice and Arabidopsis, the ABA mediated stress response in soybean under flooding stress is independent of the SUB1 pathway. Another important aspect highlighted here is the reduced overall metabolic activity under flooding stress, which supports the quiescence rather than the escape mechanism.

The resource of DEGs and also the validated expression of key genes featured in various metabolic pathways under drought and flooding stress will be helpful in understanding the complex mechanisms of stress tolerance. The study further substantiates and elaborates the affected photosynthetic capacity and sugar metabolism under these stresses in soybean. In addition, an in-depth insight into the TFs associated with drought and flooding responsive genes points toward a more complex and intricate gene regulatory network. In particular, genes encoding for LHC, sugar transporters, and the cell wall composition were identified to play a crucial role in combating stress. These genes could be targeted as potential candidates for functional validation and can also be considered for study in other legumes affected by similar stresses.

## Author contributions

WC, QY, GP, and GA are all equal contributing authors for this manuscript. WC, BV, HTN designed the experiment. QY, GP, GA, RD, LL, DX, YCA, performed bioinformatics analysis. WC, GP, GA, and RD performed data mining, analysis and interpretation. RD, GP, and GA contributed to drafting the manuscript. WC, BW, SP, and LS performed greenhouse experiment, tissue collection, RNA isolation and qRT-PCR analysis. YW constructed RNAseq library. RKV and HTN conceived the study and edited the manuscript. All authors read and approved the final manuscript.

### Conflict of interest statement

The authors declare that the research was conducted in the absence of any commercial or financial relationships that could be construed as a potential conflict of interest.

## Supplementary material

Supplementary Figure 1 **RNA-Seq analysis workflow for drought and flood stressed soybean leaf samples**.

Supplementary Figure 2 **Scatter plots of differentially expressed genes under (A) drought (B) flooding stress**.

Supplementary Figure 3 **Tetrapyrrole under (A) drought and (B) flooding**. Under flooding stress, this pathway is down-regulated.

Supplementary Figure 4 **The sucrose starch synthesis pathway under (A) drought and (B) flooding**.

Supplementary Figure 5 **DEGs categorized in different metabolic functions**. Up- and down-regulation of genes under **(A)** drought and **(B)** flooding are represented by colored squares.

Supplementary Figure 6 **DEGs categorized and mapped in primary metabolic pathways under (A) drought and (B) flooding conditions in soybean**. The log_2_ fold change value of a DEG is represented by colored squares.

Supplementary Figure 7 **The cell wall precursors synthesis pathway under (A) drought and (B) flooding**.

Supplementary Table 1 **List of genes with confident expression in drought stressed leaf tissue compared to non-stressed control tissue. **(B)** List of genes with confident expression in flooding stressed leaf tissue compared to non-stressed control tissue**.

Supplementary Table 2 **List of DEGs in, (A) drought, (B) flooding stressed leaf tissue compared to non-stressed control tissue. (C)** List of DEGs in flooding stressed leaf tissue compared to drought stressed leaf tissue.

Supplementary Table 3 **Significant GO terms among (A) up-regulated genes under drought; (B) down-regulated genes under drought; (C) up-regulated genes under flooding, and (D) down-regulated genes under flooding conditions**.

Supplementary Table 4 **(A)** Overrepresented motifs from the significantly changed flooding and drought gene sets. **(B)** Common motifs shared by flooding and drought response genes.

Supplementary Table 5 **Primers used in the qRT-PCR analysis**.
