# Supplementary figures and images for: Identification and Comparative Analysis of Differential Gene Expression in Soybean Leaf Tissue under Drought and Flooding Stress Revealed by RNA-Seq
Source: Front Plant Sci. 2016 Jul 19;7:1044. doi: 10.3389/fpls.2016.01044 (PMC4950259; doi:10.3389/fpls.2016.01044)

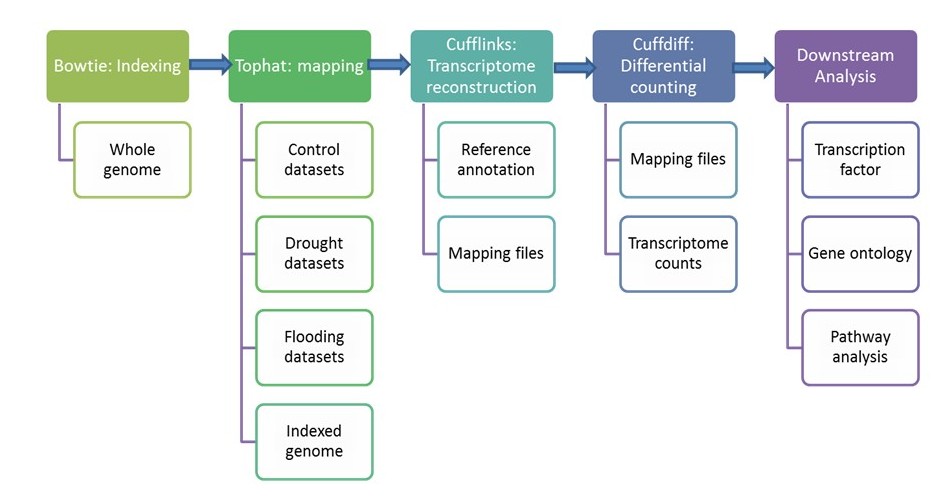

Supplement: Supplementary Figure 1 — RNA-Seq analysis workflow for drought and flood stressed soybean leaf samples. [file Image1.JPEG]

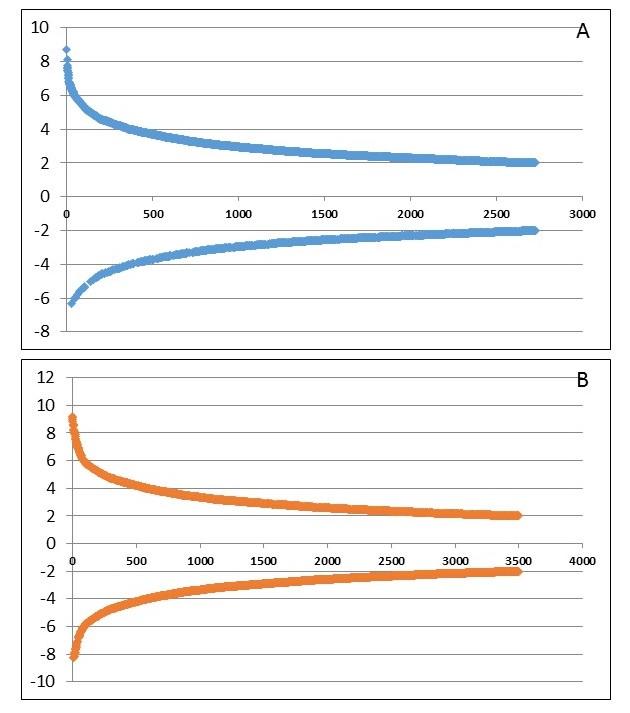

Supplement: Supplementary Figure 2 — Scatter plots of differentially expressed genes under (A) drought (B) flooding stress. [file Image2.JPEG]

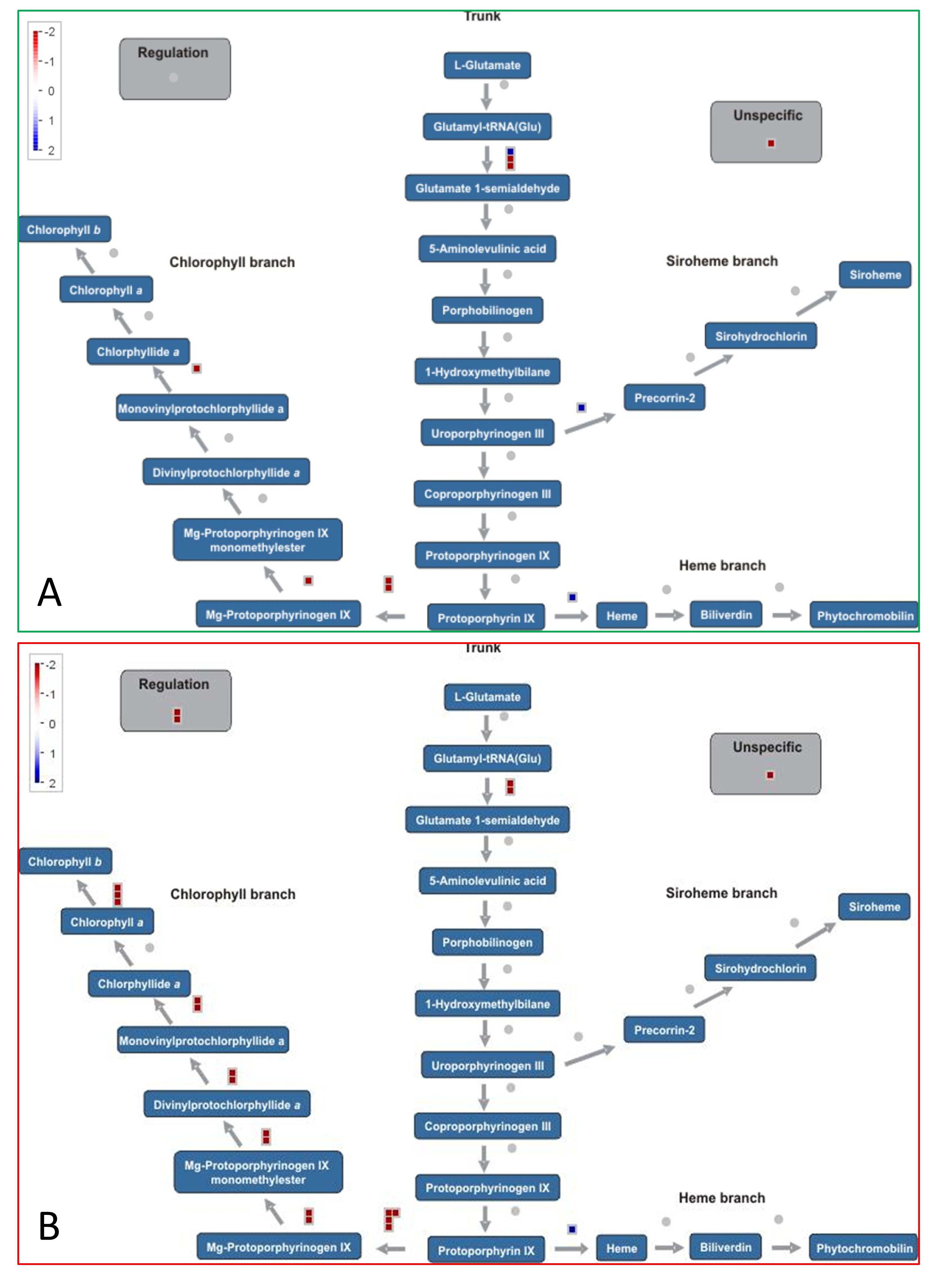

Supplement: Supplementary Figure 3 — Tetrapyrrole under (A) drought and (B) flooding. Under flooding stress, this pathway is down-regulated. [file Image3.JPEG]

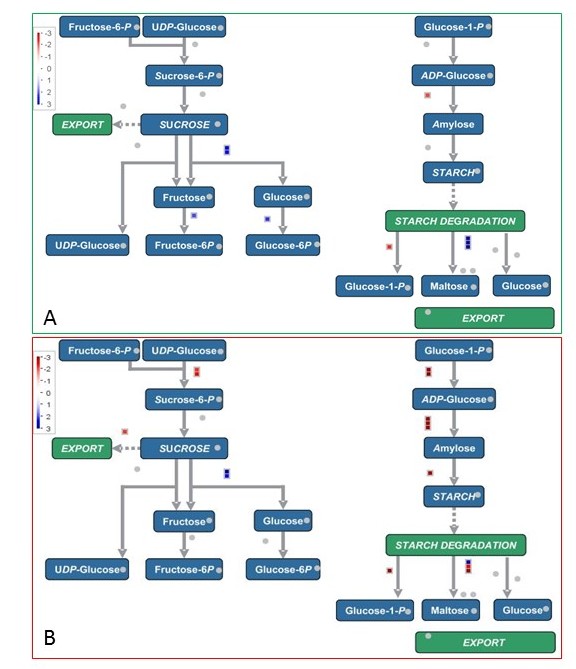

Supplement: Supplementary Figure 4 — The sucrose starch synthesis pathway under (A) drought and (B) flooding. [file Image4.JPEG]

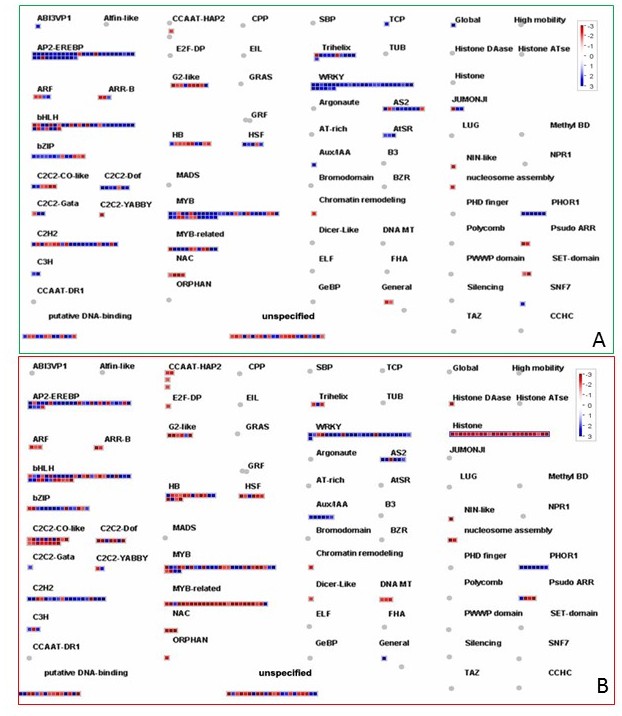

Supplement: Supplementary Figure 5 — DEGs categorized in different metabolic functions. Up- and down-regulation of genes under (A) drought and (B) flooding are represented by colored squares. [file Image5.JPEG]

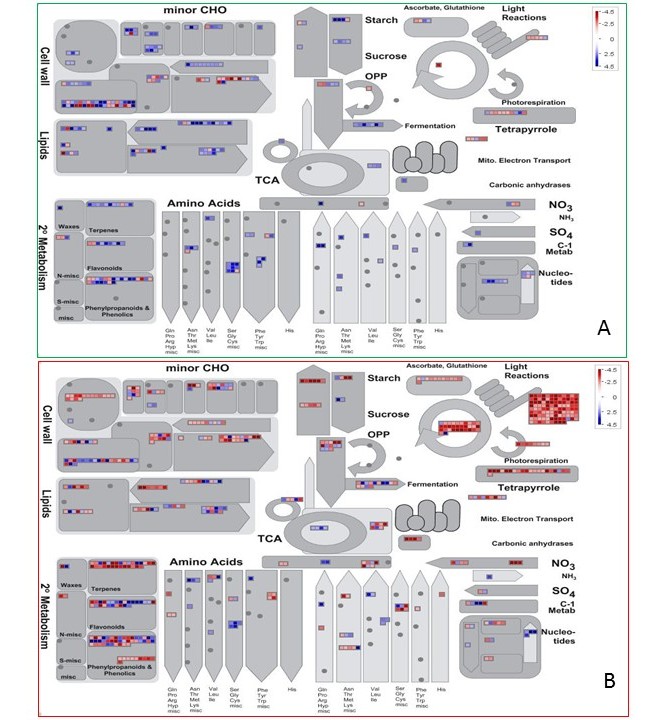

Supplement: Supplementary Figure 6 — DEGs categorized and mapped in primary metabolic pathways under (A) drought and (B) flooding conditions in soybean. The log2 fold change value of a DEG is represented by colored squares. [file Image6.JPEG]

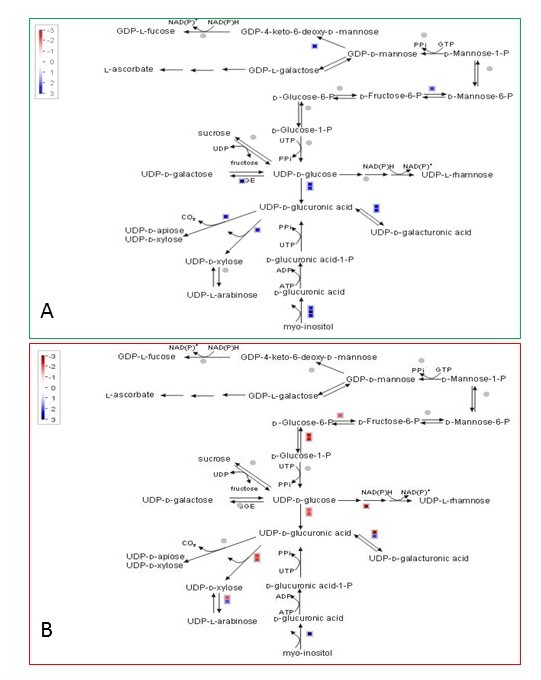

Supplement: Supplementary Figure 7 — The cell wall precursors synthesis pathway under (A) drought and (B) flooding. [file Image7.JPEG]
