# Supplementary material for: Identification and Comparative Analysis of Differential Gene Expression in Soybean Leaf Tissue under Drought and Flooding Stress Revealed by RNA-Seq
Source: Front Plant Sci. 2016 Jul 19;7:1044. doi: 10.3389/fpls.2016.01044 (PMC4950259; doi:10.3389/fpls.2016.01044)
Supplement: Supplementary Table 3 — Significant GO terms among (A) up-regulated genes under drought; (B) down-regulated genes under drought; (C) up-regulated genes under flooding, and (D) down-regulated genes under flooding conditions. [file Table3.DOCX]

**Supplementary Table 3.** Significant GO terms among **A.** up-regulated genes under drought; **B.** down regulated genes under drought; **C.** up-regulated genes under flooding and **D.** down regulated genes under flooding conditions.

**Supplementary Table 3A.**

| **GO** | ***p*_value** | **Function** | **Category** |
| --- | --- | --- | --- |
| GO:0003700 | 3.74E-50 | sequence-specific DNA binding transcription factor activity | MF |
| GO:0004672 | 1.37E-30 | protein kinase activity | MF |
| GO:0003677 | 9.36E-15 | DNA binding | MF |
| GO:0004553 | 6.12E-14 | hydrolase activity, hydrolyzing O-glycosyl compounds | MF |
| GO:0016758 | 1.51E-11 | transferase activity, transferring hexosyl groups | MF |
| GO:0005506 | 2.59E-11 | iron ion binding | MF |
| GO:0003824 | 2.38E-09 | catalytic activity | MF |
| GO:0005515 | 3.69E-09 | protein binding | MF |
| GO:0047800 | 3.73E-09 | cysteamine dioxygenase activity | MF |
| GO:0016746 | 6.10E-07 | transferase activity, transferring acyl groups | MF |
| GO:0005509 | 6.18E-05 | calcium ion binding | MF |
| GO:0043531 | 1.12E-04 | ADP binding | MF |
| GO:0022857 | 1.20E-04 | transmembrane transporter activity | MF |
| GO:0016853 | 1.82E-04 | isomerase activity | MF |
| GO:0005216 | 2.32E-04 | ion channel activity | MF |
| GO:0000902 | 2.60E-04 | cell morphogenesis | BP |
| GO:0016872 | 5.06E-04 | intramolecular lyase activity | MF |
| GO:0005215 | 5.44E-04 | transporter activity | MF |
| GO:0016787 | 5.57E-04 | hydrolase activity | MF |
| GO:0004842 | 5.59E-04 | ubiquitin-protein ligase activity | MF |
| GO:0016747 | 7.19E-04 | transferase activity, transferring acyl groups other than amino-acyl groups | MF |

**Supplementary Table 3B.**

| **GO** | ***p*_value** | **Function** | **Category** |
| --- | --- | --- | --- |
| GO:0004672 | 1.28E-16 | protein kinase activity | MF |
| GO:0005506 | 1.66E-08 | iron ion binding | MF |
| GO:0016747 | 5.79E-06 | transferase activity, transferring acyl groups other than amino-acyl groups | MF |
| GO:0016758 | 6.63E-06 | transferase activity, transferring hexosyl groups | MF |
| GO:0005215 | 2.69E-05 | transporter activity | MF |
| GO:0005337 | 5.46E-05 | nucleoside transmembrane transporter activity | MF |
| GO:0003677 | 5.59E-05 | DNA binding | MF |
| GO:0016614 | 1.25E-04 | oxidoreductase activity, acting on CH-OH group of donors | MF |
| GO:0004553 | 2.27E-04 | hydrolase activity, hydrolyzing O-glycosyl compounds | MF |
| GO:0005515 | 3.29E-04 | protein binding | MF |
| GO:0030410 | 4.02E-04 | nicotianamine synthase activity | MF |
| GO:0005471 | 4.02E-04 | ATP:ADP antiporter activity | MF |
| GO:0016491 | 4.30E-04 | oxidoreductase activity | MF |
| GO:0000156 | 5.45E-04 | phosphorelay response regulator activity | MF |
| GO:0004252 | 5.67E-04 | serine-type endopeptidase activity | MF |
| GO:0015105 | 6.00E-04 | arsenite transmembrane transporter activity | MF |
| GO:0008883 | 6.00E-04 | glutamyl-tRNA reductase activity | MF |

**Supplementary Table 3C.**

| **GO** | ***p*_value** | **Function** | **Category** | |  |
| --- | --- | --- | --- | --- | --- |
| GO:0003700 | 1.78E-46 | sequence-specific DNA binding transcription factor activity | | MF | |
| GO:0004672 | 1.78E-22 | protein kinase activity | | MF | |
| GO:0004601 | 1.73E-21 | peroxidase activity | | MF | |
| GO:0005506 | 5.42E-13 | iron ion binding | | MF | |
| GO:0006952 | 1.66E-09 | defense response | | BP | |
| GO:0022857 | 2.75E-09 | transmembrane transporter activity | | MF | |
| GO:0016747 | 5.88E-08 | transferase activity, transferring acyl groups other than amino-acyl groups | | MF | |
| GO:0016746 | 3.50E-07 | transferase activity, transferring acyl groups | | MF | |
| GO:0004842 | 1.42E-06 | ubiquitin-protein ligase activity | | MF | |
| GO:0016491 | 1.79E-06 | oxidoreductase activity | | MF | |
| GO:0004553 | 4.17E-06 | hydrolase activity, hydrolyzing O-glycosyl compounds | | MF | |
| GO:0016740 | 1.19E-05 | transferase activity | | MF | |
| GO:0016758 | 2.36E-05 | transferase activity, transferring hexosyl groups | | MF | |
| GO:0031072 | 1.24E-04 | heat shock protein binding | | MF | |
| GO:0004066 | 2.16E-04 | asparagine synthase (glutamine-hydrolyzing) activity | | MF | |
| GO:0006950 | 3.27E-04 | response to stress | | BP | |
| GO:0003677 | 8.96E-04 | DNA binding | | MF | |
| GO:0047800 | 9.01E-04 | cysteamine dioxygenase activity | | MF | |

**Supplementary Table 3D.**

| **GO** | ***p*_value** | **Function** | **Category** |
| --- | --- | --- | --- |
| GO:0003677 | 1.60E-24 | DNA binding | MF |
| GO:0016491 | 1.59E-22 | oxidoreductase activity | MF |
| GO:0015979 | 2.23E-19 | photosynthesis | BP |
| GO:0016758 | 4.72E-13 | transferase activity, transferring hexosyl groups | MF |
| GO:0005506 | 5.74E-13 | iron ion binding | MF |
| GO:0005215 | 9.84E-11 | transporter activity | MF |
| GO:0005509 | 3.70E-10 | calcium ion binding | MF |
| GO:0016702 | 4.86E-09 | oxidoreductase activity, acting on single donors with incorporation of molecular oxygen, | MF |
| GO:0042578 | 4.75E-08 | phosphoric ester hydrolase activity | MF |
| GO:0016747 | 1.21E-07 | transferase activity, transferring acyl groups other than amino-acyl groups | MF |
| GO:0004332 | 1.24E-07 | fructose-bisphosphate aldolase activity | MF |
| GO:0016020 | 7.39E-07 | membrane | CC |
| GO:0016757 | 1.46E-06 | transferase activity, transferring glycosyl groups | MF |
| GO:0004553 | 1.64E-06 | hydrolase activity, hydrolyzing O-glycosyl compounds | MF |
| GO:0009512 | 3.13E-06 | cytochrome b6f complex | CC |
| GO:0003854 | 3.41E-06 | 3-beta-hydroxy-delta5-steroid dehydrogenase activity | MF |
| GO:0016788 | 5.01E-06 | hydrolase activity, acting on ester bonds | MF |
| GO:0003824 | 5.55E-06 | catalytic activity | MF |
| GO:0004672 | 8.89E-06 | protein kinase activity | MF |
| GO:0004252 | 8.90E-06 | serine-type endopeptidase activity | MF |
| GO:0008762 | 1.26E-05 | UDP-N-acetylmuramate dehydrogenase activity | MF |
| GO:0010333 | 1.74E-05 | terpene synthase activity | MF |
| GO:0016760 | 1.80E-05 | cellulose synthase (UDP-forming) activity | MF |
| GO:0003993 | 2.51E-05 | acid phosphatase activity | MF |
| GO:0016740 | 3.01E-05 | transferase activity | MF |
| GO:0016787 | 4.07E-05 | hydrolase activity | MF |
| GO:0004512 | 5.21E-05 | inositol-3-phosphate synthase activity | MF |
| GO:0004144 | 5.88E-05 | diacylglycerol O-acyltransferase activity | MF |
| GO:0003913 | 1.44E-04 | DNA photolyase activity | MF |
| GO:0006952 | 1.72E-04 | defense response | BP |
| GO:0016655 | 1.77E-04 | oxidoreductase activity, acting on NAD(P)H, quinone or similar compound as acceptor | MF |
| GO:0004176 | 2.35E-04 | ATP-dependent peptidase activity | MF |
| GO:0016984 | 2.80E-04 | ribulose-bisphosphate carboxylase activity | MF |
| GO:0005315 | 3.06E-04 | inorganic phosphate transmembrane transporter activity | MF |
| GO:0004869 | 4.40E-04 | cysteine-type endopeptidase inhibitor activity | MF |
| GO:0008168 | 5.01E-04 | methyltransferase activity | MF |
| GO:0016853 | 5.72E-04 | isomerase activity | MF |
| GO:0004089 | 7.94E-04 | carbonate dehydratase activity | MF |
