# Supplementary material for: Identification and Comparative Analysis of Differential Gene Expression in Soybean Leaf Tissue under Drought and Flooding Stress Revealed by RNA-Seq
Source: Front Plant Sci. 2016 Jul 19;7:1044. doi: 10.3389/fpls.2016.01044 (PMC4950259; doi:10.3389/fpls.2016.01044)
Supplement: Supplementary Table 5 — Primers used in the qRT-PCR analysis. [file Table5.DOCX]

**Supplementary Table 5.** Primers used in the qRT-PCR analysis.

| **Gene ID** | **Primers 5' to 3'** |
| --- | --- |
| *Glyma06g06900.1F* | TTGATTTCTCGGCTCGGTATGC |
| *Glyma06g06900.1R* | AAGCTTCACTGTGCTCCCATTC |
| *Glyma07g16810.1F* | TGGAGCCAAGAATTCCTTAACCAC |
| *Glyma07g16810.1R* | CATAGCGGGCTTTGAAGTAGGC |
| *Glyma08g41090.1F* | TGTAGAGCGTCTTGAAATGAGTGG |
| *Glyma08g41090.1R* | CTGGTATCTGCCAAAGCACCTC |
| *Glyma11g06430.1F* | ACCAACACACTTGATGGCGTCTC |
| *Glyma11g06430.1R* | GCCGAGGCTTTGGATGACAATG |
| *Glyma12g13290.1F* | TTGTAGCATCATGACTGGCAGAG |
| *Glyma12g13290.1R* | TCCAAACCCTGCTTTCACCTTC |
| *Glyma13g01120.1F* | ACATCCTTCACACCAACGTGTTC |
| *Glyma13g01120.1R* | TCCATCCACCGAGAAGACAATACG |
| *Glyma15g01500.1F* | GCTAGTGGGAAAGCTGATGTTGC |
| *Glyma15g01500.1R* | TTGAGCCAGCCCATTTAAGGTC |
| *Glyma15g03620.1F* | CTGCAATCAAGGATGGTGCAAATG |
| *Glyma15g36200.1R* | GATGGCACCAATTACACCTCCAAG |
| *Glyma17g13010.1F* | GGTCACATACCACCTGGTTTAGGC |
| *Glyma17g13010.1R* | AAGGTGGATGAGGATGAGCATGGG |
